# Clinical value of [^18^F]AlF-Thretide PET/CT and early-time-point PET acquisition in the detection and staging of prostate cancer

**DOI:** 10.7150/thno.103667

**Published:** 2025-02-26

**Authors:** Xin Cheng, Guozhu Hou, Mingshuai Wang, Shan Zheng, Wei Yao, Xuejuan Wang, Rong Zheng, Nianzeng Xing, Jingjing Zhang

**Affiliations:** 1 Department of Nuclear Medicine (PET-CT Center), National Cancer Center/National Clinical Research Center for Cancer/Cancer Hospital, Chinese Academy of Medical Sciences and Peking Union Medical College, Beijing, 100021, China.; 2 Department of Urology, National Cancer Center/National Clinical Research Center for Cancer/Cancer Hospital, Chinese Academy of Medical Sciences and Peking Union Medical College, Beijing, 100021, China.; 3 State Key Laboratory of Molecular Oncology, National Cancer Center/National Clinical Research Center for Cancer/Cancer Hospital, Chinese Academy of Medical Sciences and Peking Union Medical College, Beijing, 100021, China.; 4 Department of Pathology, National Cancer Center/National Clinical Research Center for Cancer/Cancer Hospital, Chinese Academy of Medical Sciences and Peking Union Medical College, Beijing, 100021, China.; 5 Department of Diagnostic Radiology, Yong Loo Lin School of Medicine, National University of Singapore, Singapore, Singapore.; 6 Theranostics Center of Excellence, Yong Loo Lin School of Medicine, National University of Singapore, 11 Biopolis Way, Helios, Singapore, 138667, Singapore.; 7 Clinical Imaging Research Centre, Centre for Translational Medicine, Yong Loo Lin School of Medicine, National University of Singapore, Singapore, Singapore.; 8 Nanomedicine Translational Research Program, Yong Loo Lin School of Medicine, National University of Singapore, Singapore, Singapore.

**Keywords:** [^18^F]AlF-Thretide, prostate-specific membrane antigen (PSMA), PET imaging, prostate cancer (PCa), early scan

## Abstract

**Aim:** Recent studies have demonstrated the potential of PET/CT with ^18^F-labeled ligands targeting prostate-specific membrane antigen (PSMA), as a promising method for prostate cancer (PCa) management. The aim of this study is to assess the clinical value of [^18^F]AlF-Thretide ([^18^F]AlF-PSMA-BCH) PET/CT and early time-point PET acquisition for detecting and staging PCa.

**Materials and Methods:** From November 2022 to May 2023, a total of 73 PCa patients were included in our study. Along with whole-body PET/CT conducted at a median time of 76 min (range: 59-139 min) post-injection, a single-bed pelvic early PET/CT scan was performed, starting at a median time of 187 s (range: 161-453 s) post-injection. Visual analysis of the images was performed first, followed by semiquantitative analysis of maximum standardized uptake value (SUVmax) of primary PCa lesions, metastases, bladder, and surrounding tissues on both early and routine time-point PET/CT scans.

**Results:** Among 56 non-surgical patients (either treatment-naive or after androgen deprivation therapy only) who had previously undergone conventional imaging, N staging was revised in 4 cases, and M staging in 2 cases. In 54 patients with bone scans for comparison, 111 lesions on [^18^F]AlF-Thretide PET/CT and 41 lesions on bone scans were identified as indicative of bone metastases. The median tumor-to-bladder (T/BL) ratios for primary lesions increased from 0.33 (range: 0.03-6.22) on routine time-point PET/CT to 4.66 (range: 0.24-645.00) on early time-point PET/CT. In 94.6% (53/56) of patients, the T/BL ratios were higher on early PET/CT scans than on routine time-point PET/CT scans. However, the SUVmax of surrounding tissues was found to be higher on early PET/CT scans compared to routine PET/CT scans (external iliac vessels: 8.02 ± 1.64 *vs.* 2.66 ± 0.59; inferior vesical artery branches near the prostate: 4.70 ± 1.09 *vs.* 2.30 ± 0.49; gluteus maximus muscle: 1.19 ± 0.31 *vs.* 0.80 ± 0.25). Of the 17 patients who underwent surgery prior to PET/CT, early PET/CT scans improved the detection rate of local recurrences from 2/17 to 5/17.

**Conclusion:** [^18^F]AlF-Thretide PET/CT was shown to be a valuable imaging modality in the management of patients with PCa. Early PET/CT scans can improve the detection rate of local recurrences and provide additional information for lesions that are challenging to distinguish from urinary uptake on routine PET/CT scans.

## Introduction

Prostate cancer (PCa) is a highly prevalent malignancy, with an estimated 268,490 new cases and 34,500 deaths in 2022 in the United States 1. Additionally, it is the most frequently diagnosed cancer in men, in over 50% of countries globally. Favorable clinical outcomes and prognosis for PCa patients heavily rely on accurate staging and early detection, as the 5-year relative survival rate is approaching 100% for localized disease 2. Prostate-specific membrane antigen (PSMA), also recognized as folate hydrolase I or glutamate carboxypeptidase II, is a transmembrane protein strongly overexpressed in PCa cells. The expression of PSMA is potentially associated with Gleason score and total prostate specific antigen (PSA) level 3, 4. PSMA-targeting radiotracers are now being increasingly utilized for both diagnosis and treatment of PCa 5. The detection rate of metastatic lesions in lymph nodes and bones can be significantly improved with PSMA PET imaging 6. The findings of PSMA PET are instrumental in the choice of treatment modality, in the preoperative surgery planning, and in guiding the scale of radiotherapy 7, 8. PSMA PET is particularly valuable for cases of newly diagnosed PCa with unfavorable intermediate or high risk, PSA persistence or rise after surgery or radiotherapy, and non-metastatic castration-resistant prostate cancer identified *via* conventional imaging 9-11.

[^18^F]AlF-Thretide is a novel Al^18^F-labeled PSMA ligand developed on the basis of [^18^F]AlF-PSMA-BCH, which has shown promising imaging capability for PCa 12. When compared to ^68^Ga, ^18^F demonstrates a range of specific advantages. The cyclotron production and moderate half-life (109.7 min) of ^18^F enable high yield synthesis, allowing for broader availability and distribution to other institutions. The increased positron yield and shorter positron range may also provide better image quality 13. Compared to most ^18^F-labeled radiotracers, [^18^F]AlF-Thretide is more convenient and quicker to prepare 12. In previous studies, [^18^F]AlF-Thretide PET/CT demonstrated a higher detection rate of intraprostatic tumors than [^18^F]F-DCFPyL PET/CT in per-patient analysis and a higher detection rate than [^18^F]F-PSMA-1007 PET/CT and mpMRI in per-lesion analysis 14-16.

However, most PSMA-targeting radiotracers, including [^18^F]AlF-Thretide, are limited by high background activity in the bladder, which can obscure nearby lesions 17, 18. A common approach in nuclear medicine to differentiate tissues is to perform a dual-phase scan, taking advantage of the time difference in optimal scanning time points of distinct organs. Early PET/CT scan, performed within the first few minutes post-tracer injection, has the potential to increase the detection rate of tumor lesions near the urinary bladder, as previous studies have shown that tumor-related [^68^Ga]Ga-PSMA-11 uptake occurs earlier than tracer accumulation in the urinary bladder 19-21. If the same holds true for [^18^F]AlF-Thretide, early PET/CT scan can be an effective way to overcome the high background activity in the bladder and improve the detection rate for PCa lesions.

## Materials and methods

### Patients

**Figure 1:**
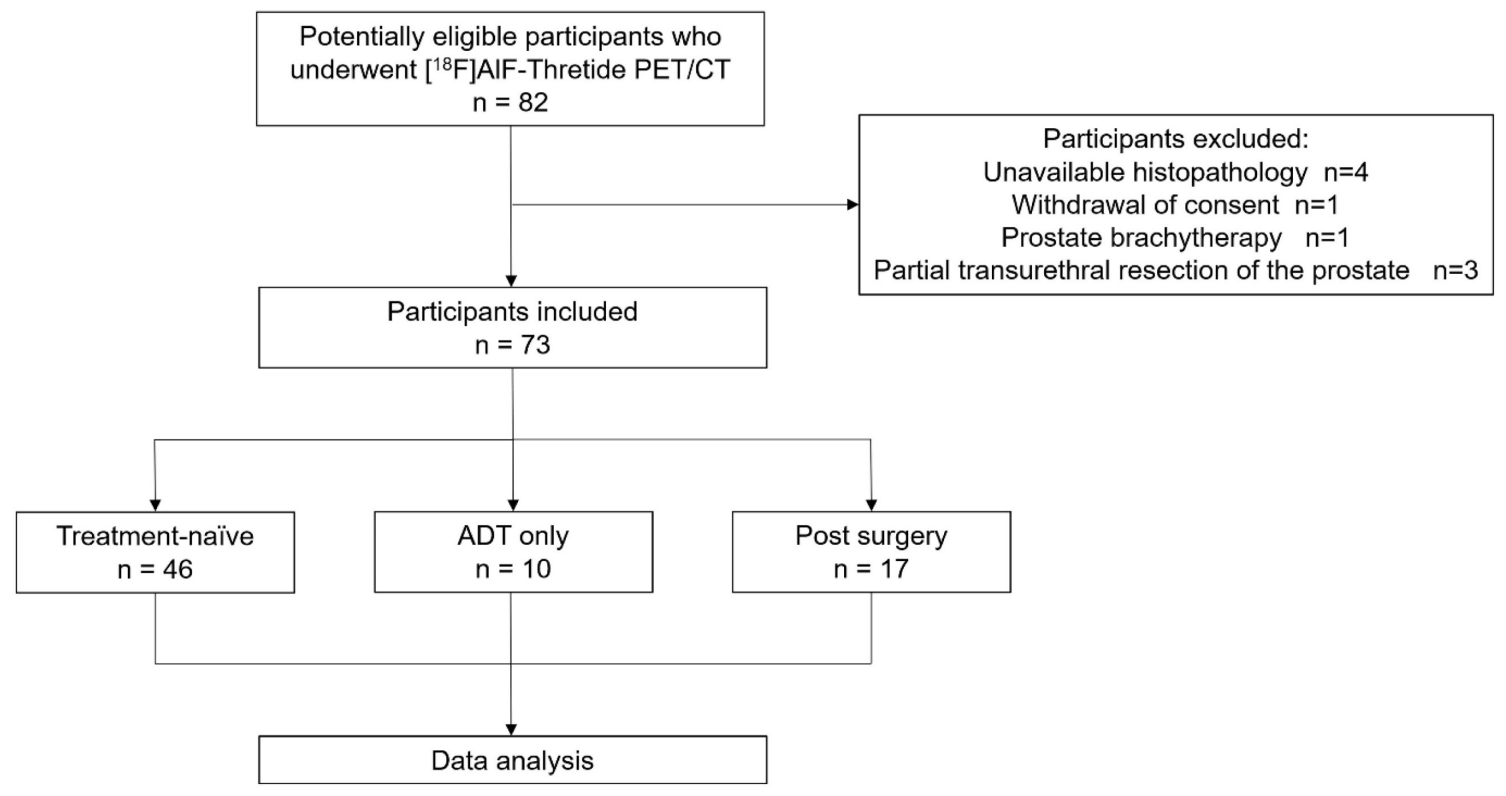
Flow Diagram.

From November 2022 to May 2023, a total of 82 PCa patients were referred to our department for participation in a clinical trial of [^18^F]AlF-Thretide PET/CT. Of these, nine patients were excluded: four due to unavailable histopathology, one due to withdrawal of consent after the scan, one due to a history of prostate brachytherapy, and three due to a history of partial transurethral resection of the prostate (**Figure 1**). Among the 73 participants included, 17 patients had undergone surgery prior to their PET/CT scans, whereas of the remaining 56 patients, 10 had received androgen deprivation therapy (ADT) and the other 46 were treatment-naive. Patient characteristics are detailed in **Table 1**.

### Radiopharmaceutical

[^18^F]AlF-Thretide was prepared using an automated synthesis module 12. The final product had a radiochemical purity of >90%, as analyzed by reversed-phase high-performance liquid chromatography and thin layer chromatography.

### Imaging protocol

Patients were injected with a mean dose of 6.37 mCi of [^18^F]AlF-Thretide (range 3.92-9.25 mCi). Single-bed early PET/CT scan was conducted using either the Discovery 690 PET/CT scanner or the Discovery MI PET/CT scanner (both from GE Healthcare), with a median acquisition starting time of 187 s (range: 161-453 s) post-injection. Early PET/CT scan was confined to the pelvic region. Subsequent whole-body routine PET/CT scan, covering from the skull base to the upper thighs, was performed on the same scanner used for the early PET/CT scan. The median acquisition starting time for routine scan was 76 min (range: 59-139 min) post-injection. Spiral CT was performed with the following parameters: 120 kV, 150 mA, pitch 1.375, 3.75 mm of slice thickness, 0.8 s of rotation speed. PET images were acquired 2 min per bed position on Discovery 690 PET/CT scanner, or 1.5 min per bed position on Discovery MI PET/CT scanner. Images were reconstructed using CT-based attenuation correction.

### Image analysis

All [^18^F]AlF-Thretide PET/CT images were analyzed using the commercially available software GE AW Server 2.0, which facilitated the review of PET, CT, and fused imaging data across axial, coronal, and sagittal slices. Any focal uptake that exceeded the surrounding background activity, and did not correspond to physiological tracer accumulation, was considered pathological, indicating the presence of malignancy. Typical diagnostic pitfalls were also taken into account.

In assessing diagnostic performance, we conducted blinded reviews of [^18^F]AlF-Thretide PET/CT, CT, bone scans, and pelvic mpMRI. For each imaging modality, two experienced readers, blinded to the results of other imaging modalities, independently reviewed the images and reached a consensus on the presence of PCa lesions within the scan range. Any disagreements between readers were resolved through consensus with a third reader.

We thoroughly analyzed all lesions within the coverage of [^18^F]AlF-Thretide PET/CT scans, including intraprostatic tumors, local recurrences, lymph node metastases, and distant metastases. The early and routine PET/CT scans were evaluated by the same set of two experienced readers. The assessment of the early scan was based on the information provided by the routine scan, and vice versa. Local recurrences, lymph node metastases, and distant metastases that were judged to be malignant were recorded and measured in size. The maximum standardized uptake value (SUVmax) was used for semiquantitative analysis of all lesions in both early scans and routine scans.

For local recurrence assessment, only lesions showing increased uptake in an identifiable area that was typical of local relapse, and distinct from urinary uptake, were considered as positive. Findings that were uncertain and could potentially indicate local recurrence but could also be attributed to urinary activity or non-tumor lesions due to their location or low uptake, were classified as negative for local recurrence.

When calculating the tumor-to-bladder (T/BL) ratios and assessing bladder radioactivity on early scans, the SUVmax of the entire urinary bladder was used if focal tracer accumulation was detected. In the absence of focal uptake, since the urinary uptake is lower than that of the bladder wall, as a secondary approach, the SUVmax for the bladder's central region was used instead, in which the region of interest was confined to a 2 cm^3^ cubic area situated at the center of the bladder.

SUVmax of the external iliac vessels was measured within a 2 cm vertical segment, centered on the upper margin of the femoral head as the reference plane. The SUVmax of the gluteal maximus muscles was measured within a 2 cm^3^ cubic area centered in the left gluteus maximus muscles at the level of the upper margin of the femoral head.

## Results

### Routine scans

**Figure 2:**
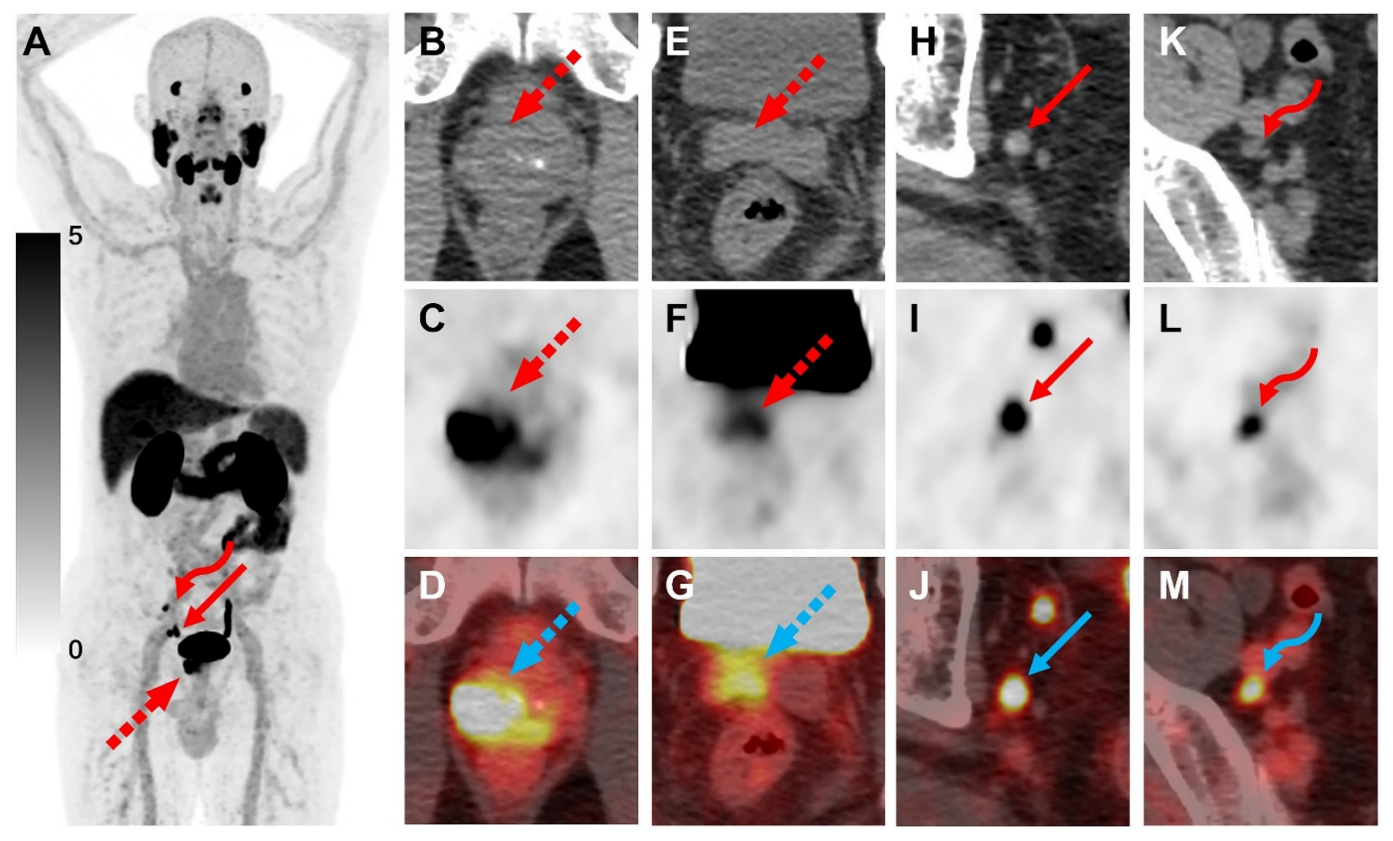
A 67-year-old man diagnosed with PCa, with a Gleason score of 4+5=9 and a total PSA level of 7.35 ng/mL, underwent [^18^F]AlF-Thretide PET/CT. **(A)** The MIP image revealed the primary PCa lesion (dashed arrow) as well as two lymph node metastases (arrow and curved arrow) in the pelvic region.** (B-G)** The scan highlighted the primary lesion's invasion into the right seminal vesicle.** (H-M)** The axial CT showed two small lymph nodes with short axes of 0.8 cm and 0.5 cm, respectively. Corresponding PET and fusion images demonstrated SUVmax of 7.9 and 10.3 for the lymph nodes. The smaller lymph node was missed by pelvic MRI. Both lymph node metastases exhibited a significant reduction in volume after ADT.

No adverse events were reported in any of the patients following the administration of [^18^F]AlF-Thretide. Of the 73 participants included, 54 had a bone scan within 4 weeks. Of the 56 non-surgical patients (either treatment-naive or who underwent ADT only), 38 had pelvic mpMRI within 4 weeks. Conventional imaging for pre-treatment staging relied on bone scans, pelvic mpMRI, and whole-body CT derived from PET/CT scans without the PET component. On routine scans, [^18^F]AlF-Thretide PET/CT exhibited excellent imaging performance for PCa lesions (**Figure 2**). Following [^18^F]AlF-Thretide PET/CT, the N or M staging was revised in six patients: five patients had their staging changed due to the detection of eight small lymph nodes with high PSMA activity (four N stage changes and one M stage change), and one patient had his M stage revised due to bone metastasis not detected by CT and bone scan. The PSA levels closest to the PET scan for these patients were 13.4, 16.7, 25.2, 54.5, 126.6 and 313.0 ng/mL. The lymph nodes that led to the staging changes had a median SUVmax of 5.27 (range: 3.65 to 21.31) and a median short-axis diameter of 5 mm (range: 4 to 6 mm). Of these, four were located along the iliac vessels, two were presacral, one was mesorectal, and one was para-aortic. These lymph nodes were missed by pelvic mpMRI and CT. All lymph nodes that led to a revision of staging were later confirmed as malignant by pathology or radiological assessments during follow-up. Among post-surgery patients, two cases were discovered to have bone metastases that were missed in bone scans, and another two cases had suspicious lymph node lesions not identified by mpMRI (with SUVmax of 2.5 and 2.8, respectively), which required follow up confirmation as the SUVs were not sufficient to confirm them as metastatic.

**Figure 3:**
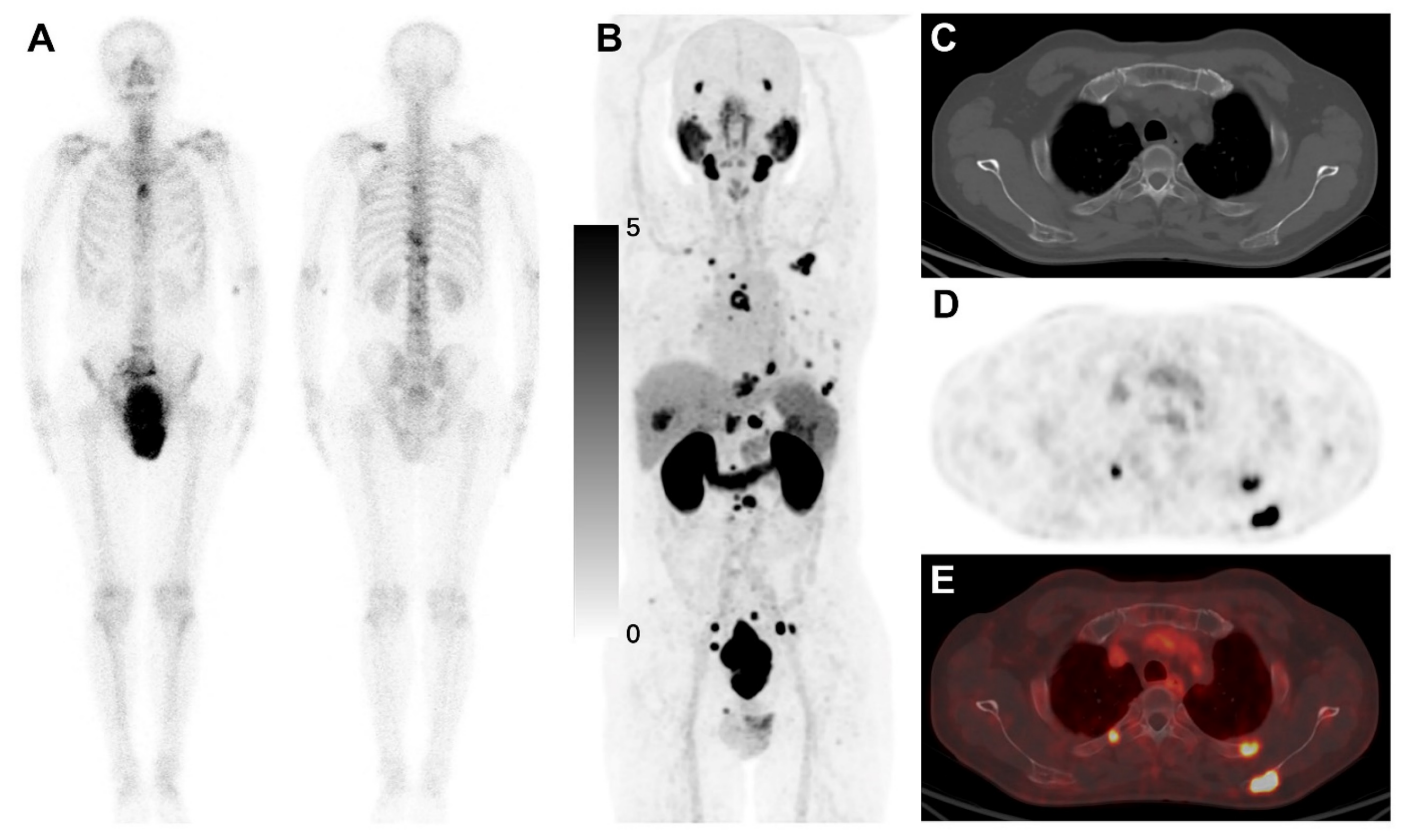
A 64-year-old man with PCa underwent a bone scan and [^18^F]AlF-Thretide PET/CT within three days. Prior to the [^18^F]AlF-Thretide PET/CT scan, his total PSA level was 540 ng/mL. **(A)** The bone scan identified 9 bone metastases. **(B-E)** However, the [^18^F]AlF-Thretide PET/CT revealed 26 bone metastases with its superior imaging capabilities. These additional lesions, not detected on the bone scan, highlight the increased sensitivity of [^18^F]AlF-Thretide PET/CT. The enhanced resolution and higher affinity of [^18^F]AlF-Thretide for PSMA-expressing tissues allowed for the identification of smaller and more subtle metastatic foci, underscoring the potential of this imaging modality to provide a more comprehensive staging in PCa.

In detecting bone metastases, [^18^F]AlF-Thretide PET/CT identified 111 bone metastatic lesions across 14 cases, while bone scans detected only 41 lesions in 11 cases (**Table 2**). Bone scans were based solely on planar bone scintigraphy, as none of the patients underwent whole-body SPECT/CT. Among the three patients whose bone metastases were detected on [^18^F]AlF-Thretide PET/CT but not on bone scans, one lesion was later suspected to be false positive. After 3 months of ADT, the SUVmax of the lesion only decreased from 5.6 to 4.1, with no osteoblastic skeletal repair observed. One case of atlantoaxial (C1 vertebra) metastasis was supported by mpMRI. Another case involved a pathological fracture obscured by the bladder on bone scan; the patient received ADT and radiotherapy for bone metastasis, leading to a decrease in PSA. The main reasons for bone scans missing bone metastases include urinary uptake obscuring pelvic metastases, benign uptake in the jaw interfering with the detection of cervical metastases, smaller lesions not showing significant changes on bone scans, and difficulties in counting lesions due to overlapping on plain radiographs (**Figure 3**). There were 29 bone metastases detected on [^18^F]AlF-Thretide PET/CT that were not visible on either bone scans or whole-body CT. Their median SUVmax was 4.71 (range: 2.94 to 11.61); median of the mean Hounsfield units across lesions was 226 (range: 80 to 747); and median long-axis diameter was 9 mm (range: 6 to 17 mm). The most common site was the spine (44.8%, 13/29), followed by the ribs (20.7%, 6/29).

### Early PET/CT scan

On early scans, compared to routine scans, urinary uptake was lower, while uptake in the circulatory system and gluteus maximus muscles was higher (**Table 3**).

**Figure 4:**
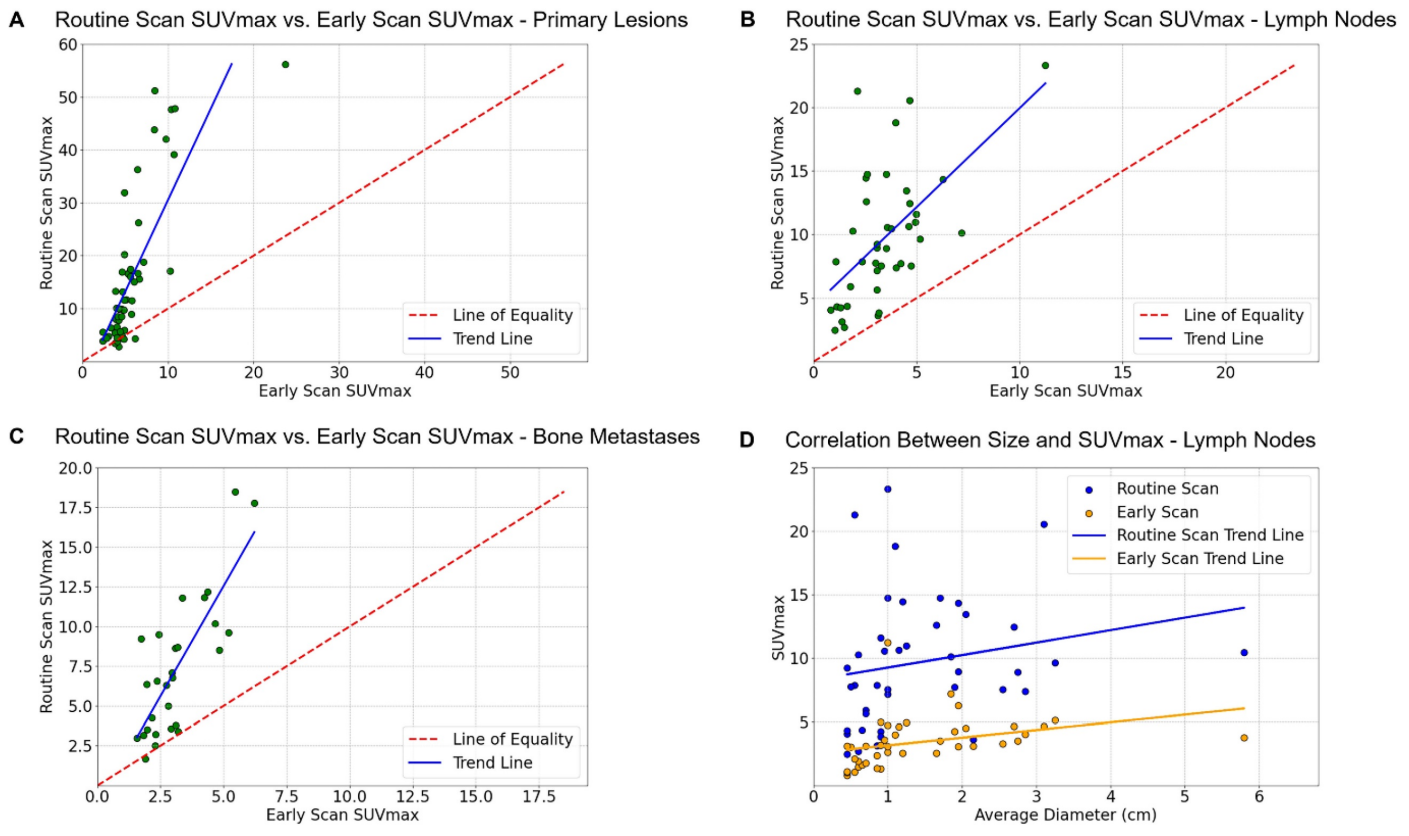
**(A-C)** On routine scans, the uptake of primary lesions, lymph node metastases, and bone metastases was generally higher than on early scans. The uptake of PCa lesions on early scans positively correlated with their uptake on routine scans. **(D)** The correlation between SUVmax and the size of lymph node metastases in early/routine scans is depicted. While uptake was positively correlated with the size of lymph node metastases, we observed that even very small lymph node metastases could exhibit high uptake.

**Figure 5:**
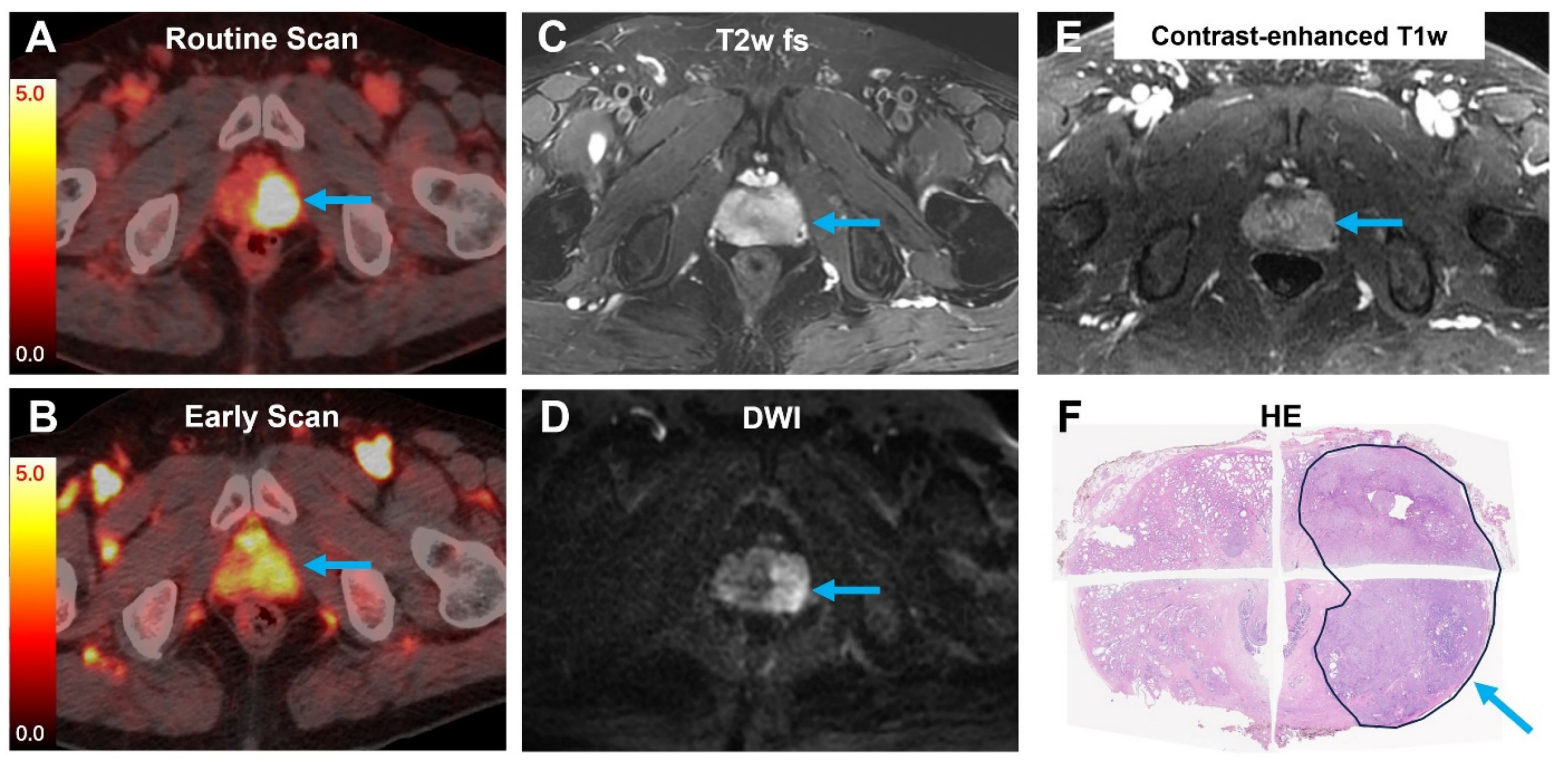
A 71-year-old man with PCa underwent pelvic MRI and [^18^F]AlF-Thretide PET/CT. His total PSA level was 21.0 ng/mL prior to the PET/CT scan. **(A-B)** Both the routine PET/CT scan and the early PET/CT scan showed intense [^18^F]AlF-Thretide uptake in the left lobe of the prostate (arrow, SUVmax 17.0 on the routine scan, 4.6 on the early scan). **(C-E)** MRI revealed a lesion with low T2 signal intensity, high DWI signal intensity, and early enhancement on the contrast-enhanced T1-weighted sequence. The lesion's margins were indistinct across all these MRI sequences.** (F)** The extent of the intense [^18^F]AlF-Thretide uptake was consistent with postoperative pathology results.

On routine scans, the uptake of prostate lesions, metastatic lymph nodes, and bone metastases were generally higher than on early scans; however, uptake in lesions during early scans positively correlated with uptake on routine scans (**Figure 4**). While the majority of lesions matched between the two scans (**Figure 5**), we occasionally observed mildly elevated activity in the transitional zone on early scans.

On early scans of the 73 patients, 43 showed no obvious radioactivity in the bladder, and 30 had focal urinary uptake that did not fill the bladder. On routine scans of 56 treatment-naive/ADT only patients, urinary uptake obscured the primary PCa lesions in 19 patients. Of these, 3 patients had their primary PCa lesions hindered by urinary uptake on early scans as well, but the remaining 16 (16/19) patients had a clearer delineation between primary PCa lesions and the bladder on early scans. The SUVmax of primary PCa lesions showed no statistically significant differences between the ADT only patients and the treatment-naive patients in either the early or routine scans.

Among 56 non-surgical patients, 34 showed no obvious radioactivity in the bladder. For these 34 patients, the median T/BL ratio on early scans was 13.02, ranging from 3.64 to 645.00, while on routine scans, it was 0.41, ranging from 0.03 to 6.22. Thus, for these patients, the T/BL ratios on early scans were 2.75 to 459.28 times higher than on routine scans. For the remaining 22 patients with focal urinary uptake in the bladder, the median T/BL ratio on early scans was 0.75, with a range of 0.24 to 4.06, while on routine scans, it was 0.32, with a range of 0.08 to 2.45. Compared to routine scans, the T/BL ratios on early scans for these 22 patients were variously higher, ranging from 0.18 to 7.77 times. In three cases, the early scans had T/BL ratios lower than those on routine scans due to focal urinary uptake, with those early scans starting 220 to 453 s post-injection. Overall, the median T/BL ratio for primary lesions increased from 0.33 (ranging from 0.03 to 6.22) on routine scans to 4.66 (ranging from 0.24 to 645.00) on early scans.

**Figure 6:**
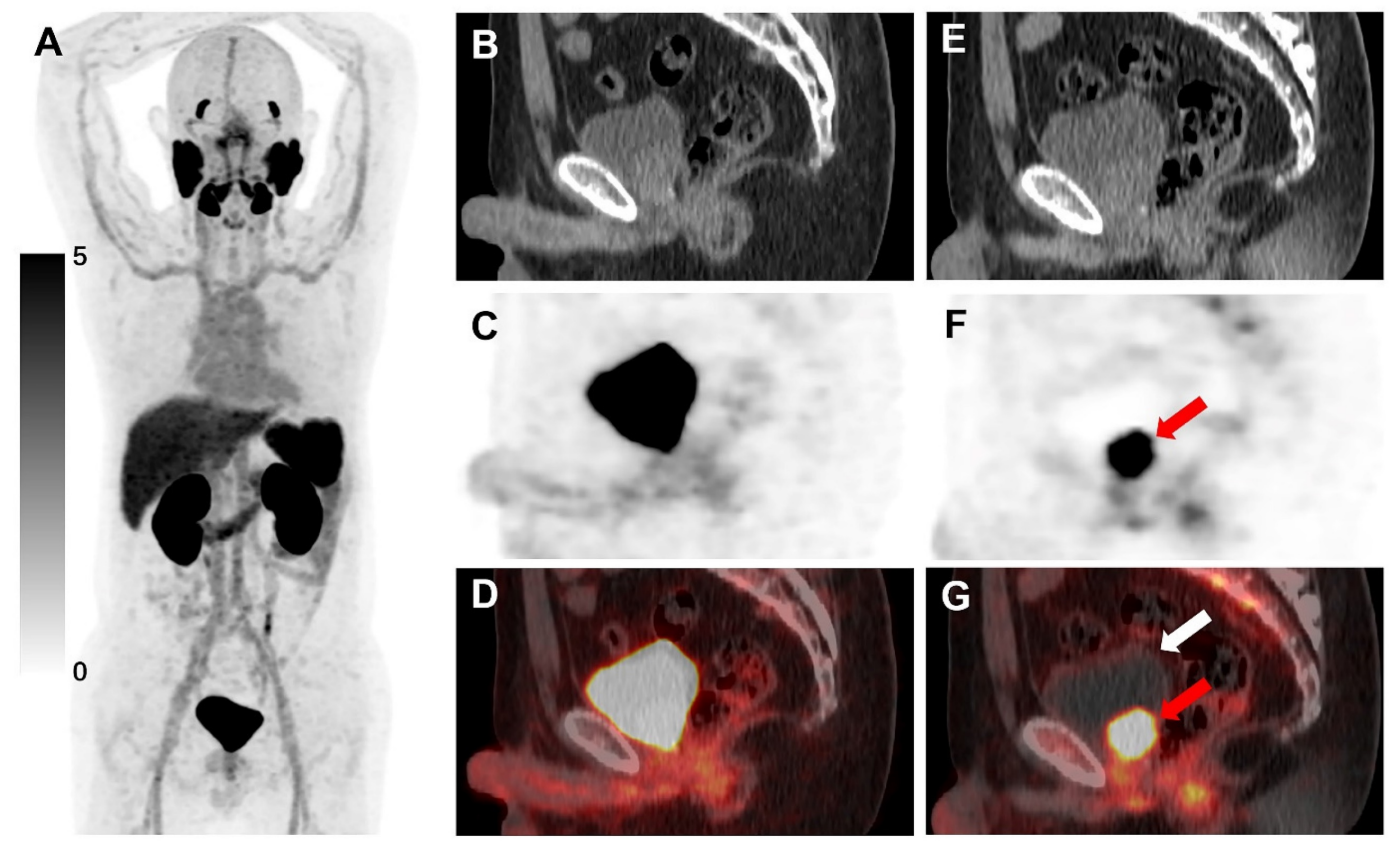
[^18^F]AlF-Thretide PET/CT images were acquired from a 54-year-old man with PCa who experienced biochemical recurrence after radical prostatectomy. His total PSA level was 0.26 ng/mL before the PET/CT scan. **(A-D)** The routine PET/CT scan conducted 60 min post-injection showed that the SUVmax of the local recurrence (SUVmax 38.1) was higher than that of the bladder (SUVmax 21.7). In our study, this local recurrence was not considered obscured by urinary uptake on the routine scan. However, tracer accumulation in the bladder would have increased the likelihood of missing this lesion in clinical practice. **(E-G)** The early PET/CT scan, conducted 182 s post-injection, clearly showed this local recurrence with significant tracer uptake (red arrow), compressing the urinary bladder. No tracer accumulation was observed in the urinary bladder at this time (white arrow). This local recurrence showed a significant reduction in volume after radiotherapy.

**Figure 7:**
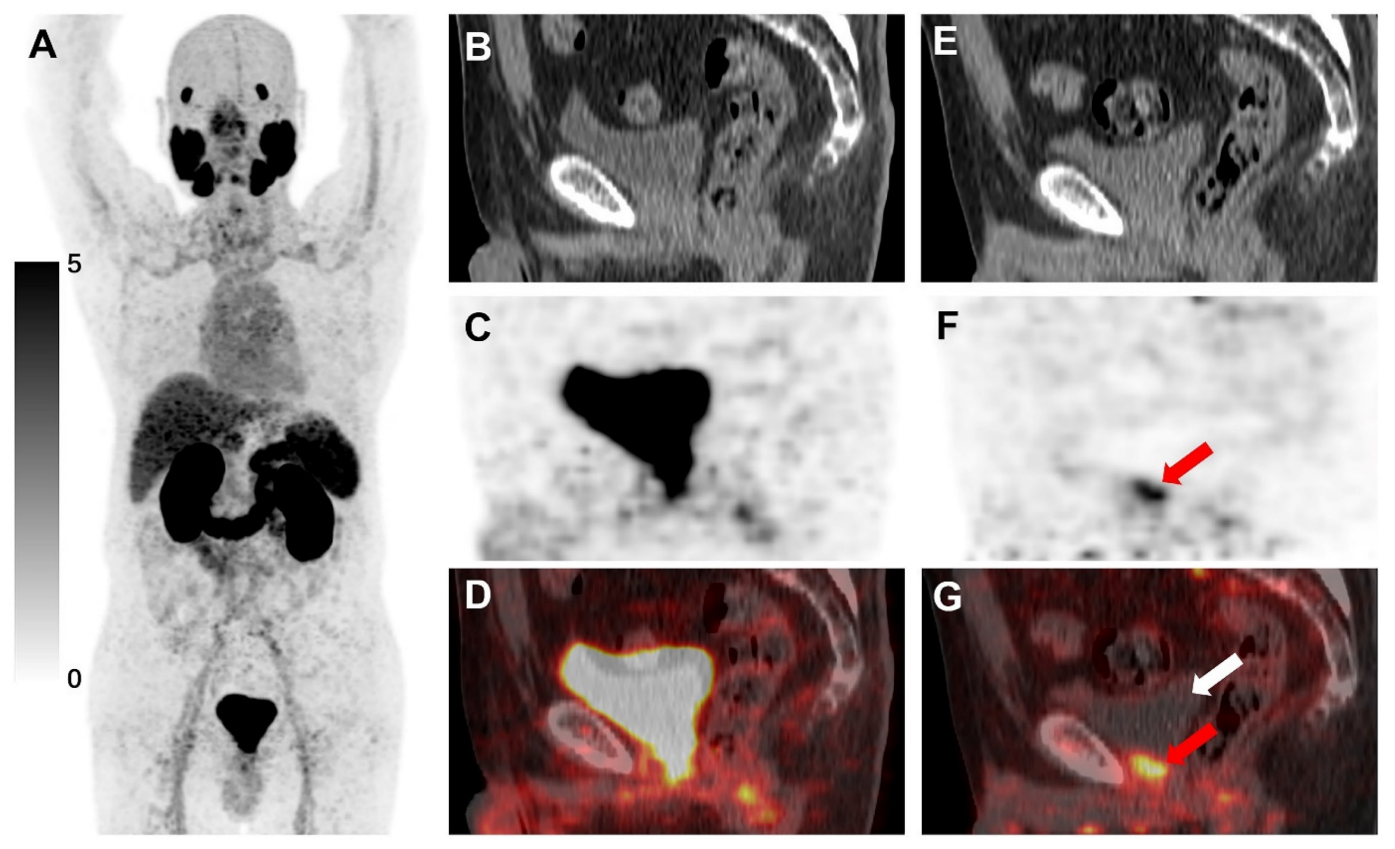
[^18^F]AlF-Thretide PET/CT images were acquired from a 64-year-old PCa patient with biochemical recurrence after radical prostatectomy. His total PSA level was 4.16 ng/mL one day prior to the PET/CT scan. The images showed focal tracer accumulation at the vesicourethral anastomosis site. **(A-D)** This accumulation closely resembled the urinary uptake in the routine PET/CT scan conducted 73 min post-injection, as shown in both the MIP image and the sagittal PET/CT images. **(E-G)** The early PET/CT scan, conducted 180 s post-injection, revealed a lesion in the same area with significant tracer uptake (red arrow, SUVmax 5.5). Notably, no tracer uptake was observed in the urinary bladder at this time (white arrow). A subsequent biopsy confirmed that this lesion was a local recurrence of PCa.

Out of the 17 post-surgery patients, 5 were found to have suspicious local recurrences in the surgical area. The PET analysis of one of the lesions was interfered by urinary uptake, but the lesion could still be distinguished from the bladder on CT. Another lesion (**Figure 6**) was located next to the bladder, making it highly likely to be missed. However, increasing the SUV display range revealed that the lesion's uptake on the routine scan was higher than that of the bladder. Urinary uptake obscured local lesions in the remaining three cases on routine scans, while early scans successfully detected these lesions. Of these three, one (**Figure 7**) was confirmed as local recurrence through biopsy, another exhibited typical recurrent features on mpMRI. The last case had been receiving ADT since two months before surgery. The post-surgical pathology showed a positive surgical margin. Five weeks after surgery, the patient underwent [^18^F]AlF-Thretide PET/CT and mpMRI, which suggested a likelihood of tumor residue or recurrence. However, after a further two months of continued ADT, the lesion showed no significant reduction on follow-up mpMRI.

## Discussion

PSMA is an attractive target for the diagnosis and treatment of PCa; numerous PSMA-targeting radiotracers labeled with ^68^Ga and ^18^F have been reported to be successful in PCa imaging 22. In addition to improving theoretical spatial resolution, ^18^F-labeled radiotracers can cover more patient needs with a single preparation and allow centralized production and distribution due to their higher yield and longer half-life than the ^68^Ga counterpart 23. In this study, we investigated the clinical application of [^18^F]AlF-Thretide PET/CT and early PET/CT scan in PCa patients.

PSMA PET/CT can alter the management approach for 27% of high-risk PCa patients with fewer than three distant metastases detected through conventional imaging 11. In our study, [^18^F]AlF-Thretide changed the staging in 6 non-surgical patients, all of whom are high-risk patients. [^18^F]AlF-Thretide detected more bone metastases at both the lesion and patient level than conventional bone scans, which often have limitations such as reduced sensitivity and problems with overlapping images. This finding is consistent with results using other PSMA ligands 24. Small-volume metastatic lymph nodes may show high uptake on [^18^F]AlF-Thretide PET/CT, as demonstrated in **Figure 4D**. This allows for earlier detection of metastatic lymph nodes compared to using CT and mpMRI. In this study, [^18^F]AlF-Thretide PET/CT was the basis for staging change in five cases of small-volume lymph nodes. Of these, two showed a decrease in size after treatment in imaging follow-ups, two exhibited pathological treatment responses in ipsilateral lymph nodes after neoadjuvant therapy plus surgery, and one was subject to incomplete surgical removal, resulting in persistently high PSA levels in the patient. This implies that even after routine imaging assessments, additional [^18^F]AlF-Thretide PET/CT can provide a more accurate staging. The clinical value of [^18^F]AlF-Thretide PET/CT and early scan is summarized in **Table 4**.

False positives remain a major concern in both bone scans and PSMA PET. To address ambiguous lesions without a biopsy, comparison with previous imaging or long-term follow-up is often necessary. Unspecific bone uptakes can occasionally be observed on PSMA PET scans, especially in the ribs 25. However, they rarely indicate bone metastases 26. Reporting physicians can adjust for these unspecific bone uptakes to avoid over-staging, either through their clinical experience or by utilizing effective predictive models 27, 28. We did not identify these lesions as positive in our study.

Both ^68^Ga and ^18^F-labeled PSMA radiotracers encounter issues where urinary uptake affects the observation of nearby lesions. Most ^68^Ga-labeled and ^18^F-labeled PSMA PET scans are performed 1 h after the tracer is administered 29. However, according to previous studies, early scans within 5 min after injection may reduce the issue of bladder interference in [^68^Ga]Ga-PSMA-11 PET/CT 19-21. To date, there are no reports on early scans of [^18^F]AlF-Thretide PET/CT.

PCa lesions typically exhibit sufficient uptake of [^68^Ga]Ga-PSMA-11 as early as 3-4 min post-injection 20. To prevent an increase in radiation dose to staff and align with future clinical scenarios, we did not adopt the ideal model of bedside injection or dynamic scanning. Instead, injections were performed in a fully shielded injection room, and patients were required to move approximately 20 meters to reach the scanner after the injection. This protocol is feasible in the majority of medical institutions. Early scans often nearly eliminate bladder radioactivity, and in cases where focal uptake remains, it typically causes minimal interference with the observation of nearby lesions. Additionally, we observed that holding urine before the examination can dilute the small amount of tracer that enters the bladder.

In a previous study, the accuracy of PSMA PET in detecting seminal vesicle infiltration was 86% and the accuracy in detecting extension beyond the capsule was 71% 30. Both PSMA PET/CT and mpMRI have comparable accuracy in detecting and localizing PCa lesions within the prostate. In visual assessment, mpMRI outperforms PSMA PET/CT in identifying extraprostatic disease 31. However, quantitative PSMA parameters are equally effective in predicting extraprostatic disease compared to the longest capsular contact measured on mpMRI 32. In our study, we found that urinary uptake during early scans was significantly lower than that during routine scans, leading to an increase in the T/BL ratios and an improved detection rate of local recurrences. Furthermore, early scans reduced urinary uptake's interference to the T staging of primary PCa lesions. Although the SUVmax of PCa lesions was lower on early scans, the enhanced T/BL ratio is likely to improve the accuracy of staging. In our study, the acquisition starting time of routine scans have a relatively wide range due to occasional delays in the scanning process. 89% (65/73) of enrolled patients started the routine scan within 100 min. Thanks to the longer half-life of ^18^F, [^18^F]AlF-Thretide PET/CT provided good image quality even with an acquisition starting time of 150 min 14.

When calculating T/BL ratios, the SUVmax of the entire bladder is taken into consideration. However, urine uptake is often uneven on early scans, resulting in lower uptake in most areas of the bladder. This is especially true when patients hold their urine before early scans. Focal urinary uptake may not necessarily be around the lesion, which means that patients with lower T/BL ratios can still benefit from early scans.

There are other PSMA targeted tracers with less urinary excretion; however, they typically result in increased uptake in the hepatic, biliary, and intestinal regions 33. Delayed post-diuretic PET/CT prolongs the examination time, but it does not completely eliminate interference from urinary uptake 34. Artificial intelligence algorithms can effectively identify lymph node metastases 35, but avoiding interference from urinary uptake remains a significant challenge. Early scans are very convenient for both patients and medical staff, incur no additional drug costs, do not prolong patient waiting times, and are highly cost-effective and feasible. Early scans in our study were limited to one bed position in the pelvic region, resulting in a relatively small increase in radiation exposure compared to the 6-8 bed positions of a whole-body PET/CT scan.

We observed that some prostates showed increased uptake primarily in the transitional zone on early scans. This phenomenon may be related to the increased blood supply associated with prostatic hyperplasia. On early scans, there was higher uptake in the circulatory system and gluteus maximus muscles compared to routine scans (**Table 3**), while the uptake of tumor lesions is lower. Out of the 49 pelvic lymph node metastases, one did not exhibit increased uptake in the early scan, and seven were obstructed by vessels with high uptake. It is important to note that early scans should not be used for diagnosis in isolation, but rather serve as a complement to routine scans.

## Conclusion

In this study, [^18^F]AlF-Thretide PET/CT was shown to be a valuable imaging modality in the management of patients with PCa. Early [^18^F]AlF-Thretide PET/CT scan can improve the detection rate of local recurrences, and provide additional information for lesions that are challenging to distinguish from urinary uptake on routine scans. We recommend integrating early PET/CT scan into the standard acquisition protocol of [^18^F]AlF-Thretide PET/CT for post-surgery PCa patients. For initial staging, early PET/CT scans also present a promising and cost-effective option, especially in patients with lesions near the bladder, or when further evaluation of the relationship between PCa lesions and the bladder is required.

## Figures and Tables

**Table 1:** Patient characteristics (*n* = 73)

Characteristic	Summary
Number of patients	73
Treatment-naive	46
ADT only	10
Post-surgery	17
Age in years, median(range)	66 (47-79)
Total PSA ng/mL, median(range)^a^	15.5 (0.0-1972.0)
Treatment-naive	23.6 (1.2-1972.0)
ADT only	12.6 (2.1-23.4)
Post-surgery	0.3 (0.0-220.8)
Gleason score^b^	
6	9 (12.3%)
3+4	5 (8.2%)
4+3	7 (9.6%)
8	26 (35.6%)
9	20(26.0%)
10	6 (8.2%)

**^a^**13 patients did not have PSA results within 4 weeks: 6 were treatment-naive, 5 had only received ADT, and 2 had undergone radical prostatectomy. **^b^**Gleason scores were determined based on best estimates, primarily using surgical pathology and secondarily using biopsy pathology. Abbreviations: ADT, androgen deprivation therapy; PSA, prostate-specific antigen.

**Table 2:** Comparison of [^18^F]AlF-Thretide PET/CT and bone scan in the detection of bone metastases.

	Imaging modality		
Parameter	[^18^F]AlF-Thretide PET/CT	Bone scan	p
Number of bone metastases*	111	41	0.007^a^
Detection rate**	14/54	11/54	0.250^b^

*In one patient with bone metastases, both [^18^F]AlF-Thretide PET/CT and bone scan showed diffuse skeletal metastases throughout the body, and the number of bone metastases was not calculated. **In 54 patients with bone scans for comparison, [^18^F]AlF-Thretide PET/CT identified bone metastases in 14 cases, while bone scans detected bone metastases in 11 cases. ^a^p value from the Wilcoxon test for paired observations tests for differences in the number of bone metastases detected between [^18^F]AlF-Thretide PET/CT and bone scan. ^b^p value from the McNemar test for paired observations tests for differences in the detection rate at the patient level between [^18^F]AlF-Thretide PET/CT and bone scan. Abbreviations: PET/CT, positron emission tomography/computed tomography.

**Table 3:** Comparison of SUVmax values of reference tissues and pelvic PCa lesions on early scans and routine scans.

	Early Scans	Routine Scans	Sample size	p value^a^
Intraprostatic tumor	5.60 ± 3.17	15.35 ± 13.82	56^b^	<0.001
Lymph node metastasis	3.41 ± 1.91	9.70 ± 5.08	41^c^	<0.001
Bone metastasis	3.13 ± 1.22	7.38 ± 4.26	28^d^	<0.001
Bladder (central area, all patients)	1.37 ± 2.37	36.60 ± 32.53	73	<0.001
Bladder (central area, no focal uptake in early scan)	0.52 ± 0.37	40.98 ± 38.36	43	<0.001
Bladder (whole bladder, focal uptake in early scan)	6.91 ± 4.49	33.56 ± 22.69	30	<0.001
External iliac vessel	8.02 ± 1.64	2.66 ± 0.59	73	<0.001
Inferior vesical artery branches near the prostate	4.70 ± 1.09	2.30 ± 0.49	52^e^	<0.001
Gluteus maximus muscle	1.19 ± 0.31	0.80 ± 0.25	73	<0.001

^a^p value from the Wilcoxon test for paired observations tests the differences between early scans and routine scans for PCa lesions, reference tissues, and the urinary bladder. ^b^Of the 73 PCa patients included in our study, 17 had undergone prostatectomy prior to scanning. The SUVmax values are from the remaining 56 pathology-confirmed PCa patients (either treatment-naive or after ADT only), representing the maximum SUV of the whole prostate. ^c^Eight lymph nodes were excluded due to being obscured by the uptake of external iliac vessels in the early scan. ^d^Of the bone metastases identified on routine scans, only 28 were within the scan range of the single-bed pelvic early scans. ^e^Inferior vesical artery branches near the prostate could not be measured in post-surgery patients; in four non-surgical patients, the inferior vesical artery branches near the prostate were obscured by intraprostatic tumors and could not be measured. Abbreviations: PCa, prostate cancer; SUVmax, maximum standardized uptake value.

**Table 4:** Clinical value of [^18^F]AlF-Thretide PET/CT and early scan

Clinical applications	Clinical value of [^18^F]AlF-Thretide PET/CT and early scan
Evaluation of intraprostatic tumors	Routine scan of [^18^F]AlF-Thretide PET/CT demonstrated a higher detection rate of intraprostatic tumors than mpMRI 14. Early scans reduce bladder interference, significantly improving the T/BL ratio for primary lesions^a^.
Detection of metastasis	Metastatic lesions show high uptake on early scans, but it is generally lower than on routine scans. On routine scans, [^18^F]AlF-Thretide PET/CT can detect small lymph nodes with high uptake, helping to identify lesions missed by CT and mpMRI^b^. [^18^F]AlF-Thretide PET/CT detects more bone metastases compared to bone scans^c^.
Postoperative follow-up	[^18^F]AlF-Thretide PET/CT provides whole-body screening. Early scans improve the detection rate of local recurrences^d^.

^a^The median T/BL ratio for primary lesions increased from 0.33 (range: 0.03 to 6.22) on routine scans to 4.66 (range: 0.24 to 645.00) on early scans. ^b^Five patients had their N or M staging changed due to the detection of small lymph nodes with high PSMA activity. ^c^In detecting bone metastases, [^18^F]AlF-Thretide PET/CT identified 111 discrete bone metastatic lesions, while bone scans detected only 41. Diffuse bone metastases observed in one patient on both [^18^F]AlF-Thretide PET/CT and bone scan were not included in this count. ^d^Of the 17 patients who underwent surgery prior to [^18^F]AlF-Thretide PET/CT, early scans improved the detection rate of local recurrences from 2/17 to 5/17. Abbreviations: T/BL, tumor-to-bladder; PET/CT, positron emission tomography/computed tomography.

## Abbreviations

ADT: androgen deprivation therapy
CT: computed tomography
DWI: diffusion-weighted imaging
HE: Hematoxylin and Eosin
MIP: maximum intensity projection
mpMRI: multiparametric magnetic resonance imaging
PET: positron emission tomography
PCa: prostate cancer
PSA: prostate-specific antigen
PSMA: prostate-specific membrane antigen
SUVmax: maximum standardized uptake value
T/BL: tumor-to-bladder
